# Clinical application of a novel hysteroscopic LNG-IUS non-suture fixation at the uterine fundus

**DOI:** 10.3389/fmed.2025.1563888

**Published:** 2025-04-28

**Authors:** Yi Yu, Hongwei Zhang, Long Sui, Limei Chen

**Affiliations:** ^1^Hysteroscopy Centre, Obstetrics and Gynecology Hospital of Fudan University, Shanghai, China; ^2^Shanghai Key Laboratory of Female Reproductive Endocrine Related Diseases, Shanghai, China

**Keywords:** menorrhagia, adenomyosis, hysteroscopy, levonorgestrel-releasing intrauterine system, fixation

## Abstract

**Objective:**

This study aimed to explore the feasibility and clinical effects of a novel hysteroscopic levonorgestrel-releasing intrauterine system (LNG-IUS) non-suture fixation at the uterine fundus.

**Methods:**

From October 2023 to July 2024, a prospective study involving a novel hysteroscopic LNG-IUS non-suture fixation at the uterine fundus was conducted at Obstetrics and Gynecology Hospital, Fudan University. The patient’s clinical symptoms, surgical time, surgical complications, postoperative LNG-IUS expulsion, and other follow-up information were recorded.

**Results:**

A total of 31 patients were included in this study. The average uterine depth is 9.17 ± 0.67 cm. Among them, 10 cases had a history of LNG-IUS expulsion. The average surgical time is 13.0 ± 4.1 min, and the average intraoperative blood loss is 5.3 ± 3.6 mL. All patients did not experience complications such as uterine perforation, massive bleeding, fluid overload, or postoperative infection. The average follow-up time after surgery was 6.0 ± 1.8 months, and no LNG-IUS expulsion occurred. The pain assessment and mean menstrual flow postoperation were less than preoperation, and the endometrial thickness and mean uterine volume postoperation were lower than preoperation, with statistically significant differences. For patients with dysmenorrhea, the postoperative relief rate was 96.3% (26/27), and for those with excessive menstruation, the postoperative effective rate reached 96.2% (25/26). The main adverse reaction was irregular vaginal bleeding, with an incidence rate of 61.3% (19/31).

**Conclusion:**

Hysteroscopic LNG-IUS non-suture fixation at the uterine fundus is a safe and effective technique, particularly suitable for patients with dysmenorrhea, excessive menstruation, or a large uterine cavity who have previously experienced LNG-IUS expulsion. This procedure is simple and minimally invasive, has a short surgical time, has minimal bleeding, and provides rapid recovery; therefore, it is worthy of clinical application.

## Introduction

The levonorgestrel-releasing intrauterine system (LNG-IUS) is an efficient intrauterine progesterone release system introduced in 1990. When placed in the uterine cavity, the LNG-IUS releases levonorgestrel into the endometrium, where its local concentration can exceed 100 times more than that of the muscular layer, and the local concentration in the endometrium can reach more than 1,000 times that those found in serum. At present, in addition to its efficient and safe contraceptive effect, LNG-IUS shows good therapeutic effects on idiopathic menorrhagia, dysmenorrhea, and adenomyosis (1, 2). According to the consensus of the American College of Obstetricians and Gynecologists (ACOG), the guidelines of the Canadian Society of Gynecological Oncology (GOC), and the Canadian Society of Obstetrics and Gynecology (SOGC) for endometrial hyperplasia, LNG-IUS is a first-line treatment (3, 4). LNG-IUS can be used in the care and treatment of early endometrial cancer (5) and can also be used to prevent the recurrence of endometrial polyps (6); therefore, it is widely used in clinical practice.

However, the overall expulsion rate of LNG-IUS is between 9.1 and 49.4% (7). For patients with diffuse adenomyosis, 3–6 injections of a gonadotropin-releasing hormone analog (GnRH-a) can be administered before using LNG-IUS to shrink the uterus. Even with the use of GnRH-a, the displacement and expulsion of the rate of LNG-IUS reached 13.3–17.3% (8). This is one of the challenges currently faced in the clinical application of LNG-IUS. To solve this problem, in this study, we innovatively attempted to fix LNG-IUS at the fundus under hysteroscopy and found that fixing LNG-IUS at the fundus muscle layer can prevent LNG-IUS displacement and expulsion and achieved satisfactory results.

## Materials and methods

### Patients

This study is a prospective clinical research conducted at the Hysteroscopy Center of Obstetrics and Gynecology Hospital of Fudan University. The study was approved by the Ethics Committee of Obstetrics and Gynecology Hospital affiliated with Fudan University (2023–143). Patients were enrolled from October 2023 to July 2024. The inclusion criteria included any one of the following: (1) Patients with a depth of the uterine cavity larger than 8.5 cm during hysteroscopy who presented with dysmenorrhea, menorrhagia, or endometrial hyperplasia and consented to place this LNG-IUS to prevent recurrence during Transcervical Resection of Polyp (TCRP) and (2) those who have a history of LNG-IUS expulsion and request to have LNG-IUS inserted again. The exclusion criteria include the following: (1) Intraoperative hysteroscopy reveals that there is an irregular shape, fragile or atypical blood vessels, or any suspected endometrial cancer; (2) Patients with malignant diseases of the reproductive system or breast cancer under treatment. For all included patients, the following information was recorded: age, menstrual history, fertility history, transvaginal ultrasound findings, hysteroscopy-related data, and postoperative follow-up information. Written informed consent was obtained from all patients. The procedure was scheduled in the first week after menstruation was over.

### Procedures: hysteroscopic LNG-IUS fixation at the fundus

Patients can choose general anesthesia or local analgesia. With lithotomy position, the patients were routinely disinfected with the external genitalia and vagina. After confirming the position of the uterus through gynecological examination, hysteroscopists used a speculum to examine the vagina and cervical canal. A cervical forceps clamp was used to fix the cervix, and then, a probe was used to measure the depth of the fundus. Hegar was used to dilate the cervical canal to 6.5 mm. Using physiological saline as the medium for uterine dilation, the initial dilation pressure was set at 15 Kpa. We used an integrated continuous flow hysteroscope (Shenda Electrics, Jiangshan City, Zhejiang Province, China) with (outer 6.5 mm Fr) a 0-degree, 3 mm optical rod lens and a 2.2-mm operating channel through which microscissors of 5Fr can be operated. A hysteroscopy was performed to examine the uterine cavity: double cornua uteri, bilateral fallopian tube ostia, fundus, anterior and posterior walls of the uterine cavity, bilateral walls, and cervical canal. The morphology of the uterine cavity, the condition of the endometrium, and the condition of the abnormal masses in the uterine cavity were observed. After a comprehensive examination under hysteroscopy, if there are polyps or thickening of the endometrium, the polyps are removed, and the uterine cavity is curettaged. Then, hysteroscopic LNG-IUS (Bayer Healthcare Co., Ltd., National Medical Products Approval No. J20090144) fixation at the fundus is performed.

**Figure 1:**
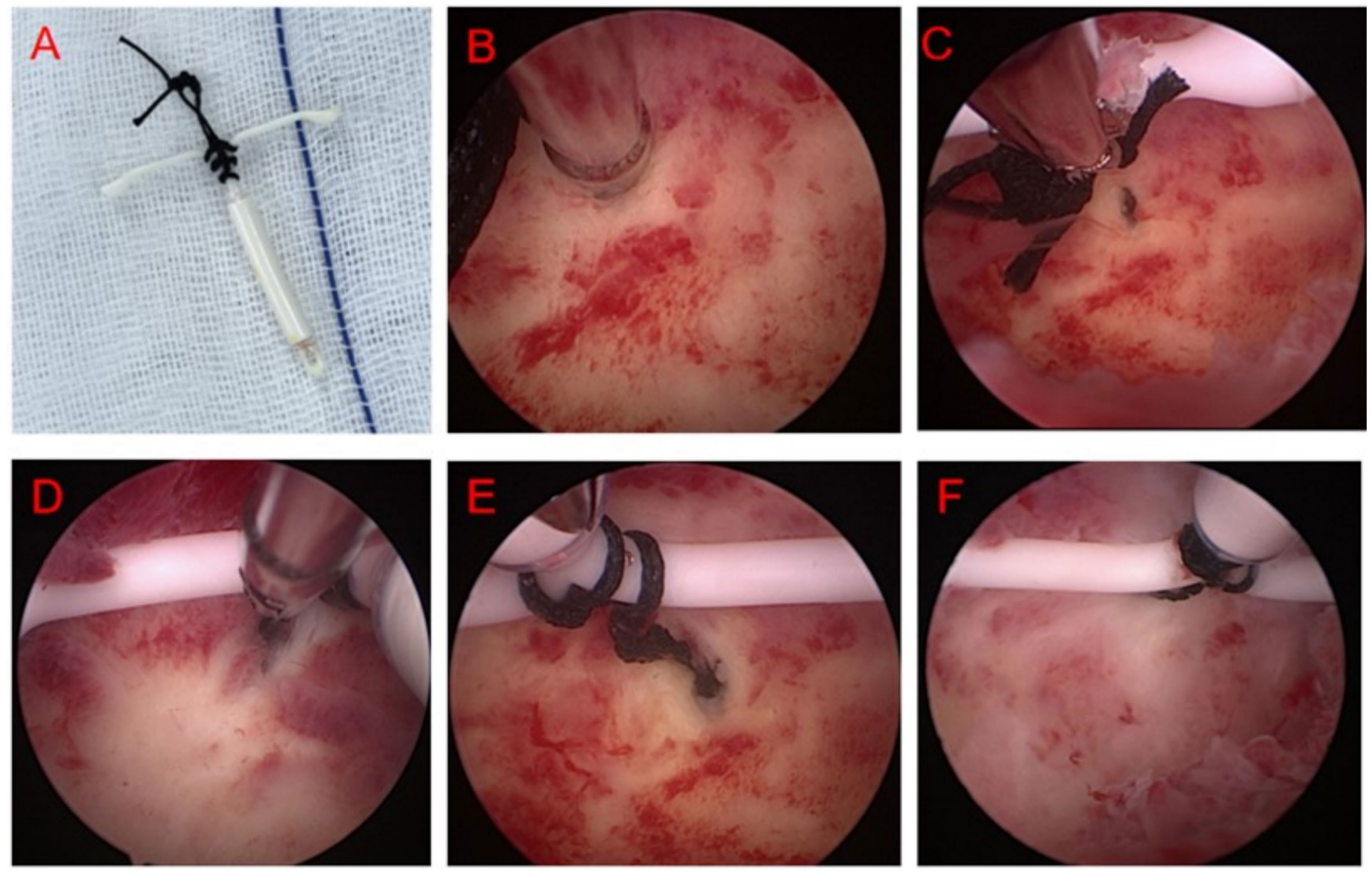
Schematic diagram of hysteroscopic fixation of LNG-IUS at the uterine fundus. **(A)** Several knots were made at the intersection of the “T” of LNG-IUS, and then, a large knot was made approximately 1 cm from the intersection of the “T”; **(B)** use micro-scissors to poke a gap of approximately 1–2 mm in the center of the fundus, with a depth of approximately 8–10 mm; **(C)** LNG-IUS with a large knot was inserted into the uterine cavity with ring forceps; **(D,E)** The large knot was inserted into the “gap” that was just formed at the fundus; **(F)** hysteroscopists withdrew the hysteroscope and massage the uterus to help anchor the knot more close to the fundus.

The specific operation procedures are as follows:

1. Several knots were made at the intersection of the “T” of LNG-IUS with non-absorbable silk thread, and then, a large knot was made approximately 1 cm from the intersection of the “T” (Figure 1A).
2. The micro tip of micro-scissors was used to poke a gap of approximately 1–2 mm in the center of the fundus, with a depth of approximately 8–10 mm (Figure 1B).
3. The LNG-IUS with a large knot was inserted into the uterine cavity with ring forceps (Figure 1C).
4. The large knot was inserted into the “gap” created at the fundus using micro scissors (Figures 1D,E).
5. After confirming that the LNG-IUS-knot would not fall off and was in the right position in the uterine cavity (Figure 1D), the hysteroscopists withdrew the hysteroscope and massaged the uterus to help anchor the knot more closely to the fundus (Figure 1F).

The general condition of the patient was continuously monitored throughout the operation, along with the difference in the inflow and outflow volumes of perfusion fluid. In addition, the operation time, bleeding volume, and any complications were recorded.

### Follow-up and efficacy evaluation

Postoperative follow-up: The main outcome of this study is focused on the expulsion of LNG-IUS. Ultrasound imaging will be performed at 1, 3, and 6 months after hysteroscopy to ascertain the position of LNG-IUS. Patients’ menstrual flow, dysmenorrhea, and satisfaction were followed up at 1, 3, and 6 months after surgery. Patients with dysmenorrhea were evaluated using the visual analog scale (VAS) before and after surgery. Patients with menorrhagia were evaluated using a pictorial blood loss assessment chart (PBAC) (9) before and after surgery. The higher the score, the greater the menstrual flow. If the score exceeds 100 points, the menstrual flow is greater than 80 mL, indicating excessive menstrual flow. If the postoperative menstrual cycle returns to normal, it is considered cured. If the menstrual flow decreases slightly but remains excessive, it is considered relieved. The relief rate = number of relieved cases/total cases * 100%, and the effective rate = (number of cured cases + number of relieved cases)/total cases * 100%. All patients underwent satisfaction surveys, and adverse reactions such as irregular bleeding, weight gain, and increased secretions were recorded.

### Statistical analysis

The data were entered in an EXCEL spreadsheet using Stata 16.0 (Stata Press, College Station, TX, US). The measurement data are expressed as mean ± standard deviation (X ± s), and a *t*-test was used for comparison before and after treatment. Count data are expressed as an example (percentage). The difference was statistically significant, with a *p*-value of <0.05.

## Result

### Characteristics of the enrolled cases

A total of 31 patients were included in this study, with an average age of 40.5 ± 4.2 years and an average uterine depth of 9.17 ± 0.67 cm (range: 8–10.5) cm. Among them, 10 cases (32.3%) had a history of LNG-IUS expulsion (7 cases had a uterine depth greater than 8.5 cm and 3 cases had a uterine depth <=8.5 cm, but the cervical canal of these three patients was relatively loose), and 21 cases had no history of LNG-IUS expulsion, but the uterine depth was all larger than 8.5 cm. Among the surgical indications, 23 patients (74.2%) had adenomyosis, of which 8 patients (25.8%) had adenomyosis with endometrial thickening. Four patients (12.9%) had endometrial polyps combined with dysmenorrhea, and three patients (9.7%) had endometrial polyps combined with menorrhagia. Two patients (6.5%) had recurrent endometrial polyps. Two patients (6.5%) had adenomyosis combined with complex endometrial hyperplasia. Regarding the setting of the procedure, 74.2% of patients (23/31) underwent outpatient hysteroscopy, while 25.8% (8/31) underwent the daily hysteroscopy. All patients were discharged from the hospital on the same day after the hysteroscopy.

### General information on LNG-IUS fixation technique under hysteroscopy

Nine patients underwent hysteroscopic endometrial polypectomy simultaneously. All patients underwent curettage before hysteroscopic LNG-IUS fixation at the fundus. The average surgical time was 13.0 ± 4.1 min, and the average intraoperative blood loss was 5.3 ± 3.6 mL. All patients did not experience complications such as uterine perforation, severe bleeding, fluid overload, or postoperative infection.

### Postoperative follow-up

The average follow-up time after surgery was 6.0 ± 1.8 months. There was no LNG-IUS expulsion found in any of the 31 patients at 1, 3, and 6 months’ follow-up. At 3 months’ follow-up, the postoperative dysmenorrhea relief rate for patients with dysmenorrhea was 96.3% (26/27), and the postoperative effective rate for patients with menorrhagia was 96.2% (25/26). The pain assessment and mean menstrual flow were lower than preoperative values, and the mean endometrial thickness and uterine volume were lower than preoperative values. The differences were statistically significant, as shown in Table 1. The main adverse reaction is irregular vaginal bleeding, with a small average volume and no further treatment. The incidence rate within 3 months after surgery is 61.3% (19/31). Seven patients experienced lower abdominal discomfort after surgery, which resolved on their own within 2–7 days without any special treatment.

**Table 1:** Comparison of VRS, PBAC, endometrial thickness, and uterine volume before and after hysteroscopic fixation of LNG-IUS in the fundus in 31 patients.

Variable	Preoperative (*n* = 31)	1 month post-operation (*n* = 31)	p*	2 months post-operation (*n* = 30)	p** (2 M VS Pre)	3 months post-operation (*n* = 28)	*p#*
VRS	4.9 ± 1.4	1.5 ± 0.6	<0.001	0.6 ± 0.4	<0.001	0.4 ± 0.3*	<0.001
PBAC	93.1 ± 28.7	71.4 ± 25.6	0.001	47.2 ± 11.5	<0.001	18.7 ± 5.8*	<0.001
Endometrial thickness	8.8 ± 2.5	6.3 ± 1.3	<0.001	4.8 ± 1.2*	<0.001	2.6 ± 0.4*	<0.001
Uterine volume (cm^3^)	141.3 ± 44.5	119.8 ± 38.3	0.023	105.7 ± 13.0*	<0.001	87.5 ± 13.5*	<0.001

*, 1-month postoperation compared to pre-operation; **, 2-months postoperation compared to pre-operation; #, 3-months postoperation compared to pre-operation.

## Discussion

### The effectiveness and challenges of LNG-IUS utilization

LNG-IUS is a highly efficient progesterone sustained-release system in the uterine cavity. In addition to its efficient and safe contraceptive effect, it has good therapeutic effects on idiopathic menorrhagia, dysmenorrhea, and adenomyosis in clinical practice. The main clinical manifestations of adenomyosis are menorrhagia and progressive aggravation of secondary dysmenorrhea, among others. The incidence rate of adenomyosis is 7–23%, with women aged 30–50 years being most likely to be affected from adenomyosis. Radical surgery, such as total hysterectomy, not only poses a short-term risk of injury and infection but also has a long-term impact on pelvic floor organ prolapse. Most women have a strong desire to preserve their uterus; therefore, conservative treatment of adenomyosis is becoming increasingly important. LNG-IUS is effective in treating dysmenorrhea, chronic pelvic pain, and menorrhagia in patients with adenomyosis and has been clinically recognized as the preferred treatment for patients with menorrhagia (10, 11). One of the challenges with LNG-IUS is its expulsion rate (7), especially in patients with adenomyosis who have an enlarged uterus and a wide uterine cavity. However, currently, the LNG-IUS is only available in one uniform size; thus, the size of LNG-IUS does not match the size of the uterine cavity, which also increases the expulsion rate. Even if GnRH-a is used in advance, the rate of LNG-IUS dislocation and expulsion remains between 13.3 and 17.3% (8). Thus, reducing the expulsion of LNG-IUS has always been of concern for clinical gynecologists and patients.

### The existing LNG-IUS fixation methods and the advantages of hysteroscopic LNG-IUS fundus fixation in this study

There are currently several reported methods to fix LNG-IUS in the uterine cavity. In 2021, Zhu et al. first reported a case (12) of a patient with adenomyosis who had a history of LNG-IUS expulsion. The LNG-IUS was sutured onto the posterior wall of the uterus using non-absorbable suture under hysteroscopy. Afterward, Paul et al. (13) and Cui and Huang (14) also published similar case reports. There are also group reports on this technique (15, 16). The above method of fixation has a definite effect, but most hysteroscopic LNG-IUS suture fixation surgery requires a large cold knife instrument, which has high requirements for hardware equipment. The equipment should dilate the cervical canal to at least 8.5 mm and generally needs to be performed under anesthesia. Moreover, hysteroscopic suture operation is not a routine operation of hysteroscopy; thus, the learning curve of this technique is long. After 5 years, the removal of LNG-IUS requires another hysteroscopic procedure to cut the suture before removal, making it only suitable to be performed in the hospital with a hysteroscope. The technique used in this study is conventional diagnostic hysteroscopy in most hospitals, using a 5Fr channel through which micro-scissors can be inserted. This instrument is relatively thin, does not require cervical dilation or anesthesia, and is more minimally invasive. It does not require hysteroscopic cold knife suturing instruments, has a short learning curve, is more minimally invasive for patients, and is more convenient for the removal or replacement of LNG-IUS. For physicians, the surgery is simpler and easier to learn. It has lower requirements for medical equipment and is more suitable for wider applications.

The technique of hysteroscopic LNG-IUS fixation at the fundus used in this study requires that the micro-scissors at the fundus not be too large, as this could easily cause the knot to fall off. Our previous research found that the average thickness of the uterine fundus in healthy women of gestational age was 10.28 ± 2.23 mm for those who were not pregnant, 11.87 ± 2.37 mm for those who gave birth to one child, and 12.26 ± 2.01 mm for those who gave birth to two or more children (17). Therefore, we found that puncturing the uterine fundus with a thickness of approximately 8–10 mm is relatively safe and does not cause perforation. In addition, the literature has reported a significant increase in the dropout rate of LNG-IUS when the uterine depth is greater than 8 cm (18). However, the expulsion rate of LNG-IUS is not completely related to the uterine depth, and it is also related to whether the cervical canal is loose. Moreover, there is currently no definition of the specific uterine depth suitable for fixation surgery.

The incidence of irregular vaginal bleeding, which is the main postoperative adverse reaction in this study, was 61.3%. This finding is similar to the 58.3% incidence of conventional LNG-IUS placement in previous studies (7). The cause of spotting bleeding is related to the local effect of LNG-IUS, and patients need to be fully informed before surgery.

There are some limitations to this study: no control group was set up during the same period, the number of cases included in this study is limited, the follow-up time is short, and the risk of long-term expulsion needs further follow-up for a longer period of time. In addition, this is a single-center study with certain selection bias, and further observation is needed in multi-center-controlled studies with larger sample sizes.

## Conclusion

In summary, the technique of hysteroscopic LNG-IUS non-suture fixation at the fundus reported in this study is novel, providing a new option for patients with adenomyosis who are prone to LNG-IUS expulsion because of a large uterine cavity and heavy menstrual flow. It has the advantages of simple operation, a short learning curve, minimally invasiveness, short operation time, fast recovery, effective fixation at the uterine fundus, and easy removal. Thus, there is clinical application value in this technology.

## Data Availability

The original contributions presented in the study are included in the article/supplementary material, further inquiries can be directed to the corresponding author.
